# Simultaneous inactivation of antibiotic-resistant bacteria and degradation of antibiotic-resistant genes in alkalised human urine

**DOI:** 10.3389/fmicb.2025.1605625

**Published:** 2025-08-22

**Authors:** Natnael Demissie, Annika Nordin, Prithvi Simha, Isis Conroy, He Sun, Anna Schnürer, Björn Vinnerås, Adey Desta

**Affiliations:** ^1^Department of Energy and Technology, Swedish University of Agricultural Sciences, Uppsala, Sweden; ^2^Institute of Biotechnology, College of Natural and Computational Sciences, Addis Ababa University, Addis Ababa, Ethiopia; ^3^Department of Environmental Engineering, Tampere University of Applied Sciences, Tampere, Finland; ^4^Department of Molecular Sciences, Swedish University of Agricultural Sciences, Uppsala, Sweden; ^5^Department of Microbial, Cellular and Molecular Biology, College of Natural and Computational Sciences, Addis Ababa University, Addis Ababa, Ethiopia

**Keywords:** fertilizer, microbial risk, safe nutrient recycling, pathogens, source separation, wastewater, hygienisation

## Abstract

The coexistence of pharmaceuticals and microorganisms in source separated urine poses a risk for the development of antimicrobial resistance (AMR), especially when urine-based fertilizers are applied to soils. While prior studies have investigated pathogen inactivation in source-separated wastewater matrices, few have evaluated the simultaneous fate of antibiotic-resistant bacteria (ARBs) and their corresponding resistance genes (ARGs) in real urine matrices, particularly under alkaline conditions. Here, we studied the inactivation of β-lactamase-producing *Escherichia coli* and vancomycin-resistant *Enterococcus faecium* and the degradation of their respective ARGs (*bla*_CTX − M_ and *van-*A) in alkalized, unhydrolyzed urine (pH 10.8 and 12.5) treated with UV (65 W low pressure dichromatic mercury lamp at 185/254 nm), hydrogen peroxide (1.25 g L^−1^ H_2_O_2_), and their combination (UV/H_2_O_2_). UV/H_2_O_2_ treatment resulted in >7 log_10_ inactivation of both ARBs, with inactivation rate constants of −0.058 log_10_ cfu min^−1^ (*E. coli*, UV) and −0.093 log_10_ cfu min^−1^ (*E. faecium*, UV/H_2_O_2_). In contrast, ARG reduction was limited with UV alone and negligible with H_2_O_2_ alone. Gene copy reductions of 3 log10 (*bla*_CTX − M_, *k* = −0.055 log10 copies min^−1^) and 2 log10 (*van-*A, *k* = −0.040 log10 copies min^−1^) were observed under UV/H_2_O_2_. Notably, brief storage (>3 h) at pH 12.5 achieved similar ARB inactivation and ARG reduction as 80 min of UV/H_2_O_2_ treatment at pH 10.8, offering a low-energy alternative for sanitizing source-separated urine.

## 1 Introduction

Urine contains valuable nutrients, such as nitrogen, phosphorus, and potassium, and around 30 million tons of nitrogen is excreted globally through urine, which can potentially replace almost 35% of the global nitrogen demand as a fertilizer (Larsen et al., 2021; STATISTA, 2024). The nutrients in source-separated urine, however, are diluted; for example nitrogen accounts for only 0.05–0.06% in urine while it is ~20% in commercial NPK fertilizer (Vinnerås et al., 2006; Senecal, 2020). For technologies aimed at recovering nitrogen from urine in the form of urea, such as urine drying, chemical stabilization is required to prevent hydrolysis of urea by the urease enzyme (Senecal and Vinnerås, 2017). Acidification (pH < 3) (Ray et al., 2018), alkalization (pH ≥10.5; Senecal, 2020), and electrochemical (Arve and Popat, 2021) methods are some approaches that are in use to stabilize urea in fresh urine, after which it can be concentrated with minimal nitrogen loss (Simha, 2021). However, there are concerns related to the presence of pathogens and micropollutants such as pharmaceuticals in source separated human urine (Bischel et al., 2015).

Consumed pharmaceuticals, such as antibiotics, end up in urine with up to 90% of their therapeutic dose (Lienert et al., 2007). Pathogens predominantly enter source separated urine through either cross-contamination with feces during collection whereas some pathogens are excreted via urine such as *Leptospira spp, Schistosomiasis haematobium* and *typhoid salmonellas* (Flores-Mireles et al., 2015). Urinary tract infection (UTI), commonly caused by fecal bacteria, will result in microorganisms excreted with the urine (Flores-Mireles et al., 2015). Skin bacteria may also be found in the urine (Schönning et al., 2002). The coexistence of microorganisms and non-lethal doses of antibiotics in urine creates conditions for the development of antimicrobial resistance (Zhou et al., 2021; Woldeyohannis and Desta, 2023). Although some regulations exist for microbial and chemical contaminants in recycled water and biosolids (e.g., *E. coli* limits in the EU; heavy metals and pathogens in the US), there are currently no regulatory limits for ARGs in source-separated wastewater fractions (WHO, 2013; Hamam et al., 2024; EPA, 2025).

Different treatment technologies, such as long-time storage (Höglund et al., 2002), alkalization (Senecal et al., 2018), ammonification (Nordin et al., 2009) and UV-based oxidation (Giannakis et al., 2018) have shown promising results for pathogen inactivation in urine. For instance, more than 6 log_10_ inactivation of *S. typhimurium* was reported in alkalized urine at pH 10.5 (Senecal et al., 2018). However, some microorganisms, such as *Clostridia* and *Salmonella spp*, have been observed to survive long periods of storage of hydrolyzed urine (36 days) by forming spores and extracellular polymeric substances (EPS), respectively (Höglund et al., 2000). In addition to ARB, antibiotic resistance genes (ARGs) can be transferred via urine. For example, (Zhou et al. 2021) reported that there were no significant changes in the concentration of the intracellular tetracycline resistant (tet M) gene after a 30 day storage in hydrolysed urine whereas (Woldeyohannis and Desta 2023) observed increase in ARGs during storage. Thus, ARGs may still persist and there is a risk of transmission downstream in the process (Zhou et al., 2021). For example, genes giving resistance against ampicillin, β-lactams, fluoroquinolone, sulphonamide, tetracycline, and vancomycin has been found in hydrolyzed urine stored for 20 days and in urine derived struvite fertilizer.

Previous research shows that electrochemical oxidation can reduce ARGs such as *bla*_KPC_ and *bla*_TEM_, with up to 4 log units in hospital urine (Herraiz-Carboné et al., 2022). However, the investigation was limited to synthetic urine and the results might differ significantly if real urine matrix is used. This is because real urine contains more than 2,500 metabolites compared to synthetic urine, which has less than 15 metabolites (Simha et al., 2024). A study on the fate of ARGs in hydrolyzed urine reported a transformation efficiency of ARGs decrease by >2 log upon incubation for 24 h (Goetsch et al., 2020). However, the study was limited to extracellular plasmid DNA whereas intracellular DNA and its inherent ARGs can survive long storage (30 day) in hydrolyzed urine (Zhou et al., 2021).

With the exception of studies on long-term storage of hydrolyzed urine (Zhou et al., 2021), the simultaneous inactivation of pathogens and the fate of antibiotic-resistant genes (ARGs) in real source separated urine have, to our knowledge, not been studied, nor in alkalized urine. Alkalization of urine produces a harsh environment for microorganisms as well as other biological material and may prevent development and exchange of antimicrobial resistance and potentially degrade ARGs (Nordin, 2010). Inactivating the resistome (ARBs and ARGs) prior to application on agricultural land will promote a safe recovery of nutrients from urine.

UV treatment has shown potential in degrading pharmaceuticals including antibiotics (Demissie et al., 2023), inactivate enzymes (Demissie et al., 2024), and pathogens (Giannakis et al., 2018) in water and wastewater matrices, including source separated real human urine. UV radiation damages DNA and affect cell integrity by altering aromatic amino acids that make up the bacterial cell wall, e.g., phenylalanine, lysine, histidine, and tryptophan (Cutler and Zimmerman, 2011; Howe et al., 1965; Goosen and Moolenaar, 2008). Further, low wavelength UV light radiation (≈200 nm) can be absorbed by bases of DNA nucleotides (Duarte, 2015). UV emission at lower wavelength (< 200 nm) can also homolyze water to produce oxidants such as hydroxyl radicals *in situ* (Zoschke et al., 2014). Hydroxyl radicals react with DNA bases with a rate of ≥109 M-1 s^−1^ (Michaels and Hunt, 1973). UV absorbance at lower wavelength and reaction of DNA bases with OH^*^ enhances DNA degradation during UV treatment. The application of oxidisers like H_2_O_2_ in conjunction with UV radiation amplifies the inactivation of microorganisms through (i) oxidative stress induced by H_2_O_2_ and (ii) UV-mediated oxidation due to the generation of additional reactive oxygen species, such as hydroxyl radicals (OH^*^) (Rincon and Pulgarin, 2004). (Moreno-Andrés et al. 2016) reported that the use of 5 mg L^−1^ H_2_O_2_ increased pathogen inactivation in salt water by 30% compared to inactivation by UV alone.

This study investigates the inactivation of β-lactamase-producing *Escherichia coli* and vancomycin-resistant *Enterococcus faecium*, along with the degradation of their corresponding intracellular resistant genes, *bla*_CTX − M_ and *van-*A, in unhydrolysed urine stabilized with KOH. Treatments included UV irradiation, H_2_O_2_, and their combination (UV/H_2_O_2_). *Escherichia coli* and *Enterococcus faecium*, were selected for this study being (i) commensal fecal bacteria, (ii) being the most common causes of UTI (Flores-Mireles et al., 2015) and (iii) prioritized by WHO for development of new drugs due to increasing clinical resistance (WHO, 2024). Overall, this work contributes to advancing safe nutrient recycling from source-separated urine by demonstrating that chemical stabilization can simultaneously address microbial risks through ARB inactivation and ARG degradation.

## 2 Methods

### 2.1 Experimental set-up

Urine was UV irradiated in a cylindrical stainless steel photoreactor (45 cm length and 3.1 cm OD). The photoreactor was equipped with a 65 W low pressure high output mercury lamp (GPHHVA357VH, LightTech, Hungary) emitting UV light at wavelengths of 254 nm and 185 nm. The photoreactor was then placed in a chamber with running water to maintain a temperature of 21 ± 2 °C inside the reactor. The fluence rate of the lamp was measured by iodine/iodide actinometry (0.184 ± 0.005 mW cm^−2^) following the procedure as described before (Rahn, 1997). Quantum yields were calculated assuming all the light reaching iodide-iodate solution had a wavelength of 254 nm.

### 2.2 Urine collection and treatment

Urine donations (*n* = 37) were collected from both male and female volunteers (aged 20–65 years) using high-density polyethylene bottles with lids. The collected urine was pooled, dosed with 2.35 g KOH L^−1^ and mixed. The alkalized urine was then kept at room temperature (20 ± 2 °C) until further use (15–20 days).

Urine which had been alkalinized (pH 10.8) was inoculated with *Escherichia coli* and *Enterococcus faecium* to study the inactivation of bacteria by plate count and fate of their resistance genes by qPCR when subjected to treatment of UV, H_2_O_2_ or in combination (UV/H_2_O_2_). The inoculated urine was subjected to the treatments for time periods of 5, 10, 20, 40, and 80 min (Table 1) and analyzed by destructive sampling design with a single replicate per time point. As such, no technical or biological replicates were performed at individual time points. In addition to the main experiment using urine at pH 10.8, urine alkalinized to 12.5 and neutralized to pH 7.0 was studied for some time periods. Treatments at pH 7 and 12.5 were conducted as controls to test the efficacy of the treatment; fresh urine were accounted by treatment at pH 7 while treatment at pH 12.5 represents urine alkalized by strong bases such as KOH.

**Table 1:** Type of treatment and exposure times for neutral pH real urine and KOH alkalized real urine at pH 10.8 and 12.5.

**Treatment type**	**Treatment time (minutes)**		
	**pH 10.8**	**pH 12.5**	**pH 7.0**
UV	5, 10, 20, 40 and 80	5 and 20	5 and 80
H_2_O_2_	5, 10, 20, 40 and 80	5 and 20	5 and 80
UV/H_2_O_2_	5, 10, 20, 40 and 80	-	-
Control	5, 20, and 80	5 and 20	5 and 80

### 2.3 Bacteria cultivation, inoculation and enumeration

The strains used for this study were *Escherichia coli* (CCUG 62975) with *bla*_CTX − M_ gene and *Enterococcus faecium* (NCTC 12202) with *van*-A gene. Inactivation of bacteria was investigated by plate count whereas the fate of their resistance genes (*bla*_CTX − M_ and *van*-A respectively) was investigated by qPCR. The bacteria were cultivated in two steps in nutrient broth and overnight cultures (37 °C, 12h) and aliquoted into 35 ml portions for *E. coli* and 40 ml portions for *E. faecium*, respectively. The portions was then centrifuged (4,500 rpm for 10 min), and the supernatant was discarded while bacterial cells were retained in the pellet. The bacteria pellets were resuspended in 0.5 ml saline solution and refrigerated at 4 °C and used that same day. At the start of an experiment the prepared bacteria were used to inoculate 600 ml urine, resulting in a start concentration of 8 log_10_ cfu ml^−1^ and 6 log_10_ cfu ml^−1^ urine for *E. coli* and *E. faecium* respectively.

For enumeration of bacteria, 1 ml of urine was sampled before and after each treatment (Table 1). One (1) ml urine was serially diluted in Buffered saline solution with peptone and Tween 80. Cultivation of *E. faecium* strain was carried out by growing it on CHROMagar (Chromagar TM) with and without vancomycin (6 mg L^−1^), while the *E. coli* strain was grown on Tryptone Bile X-Glucuronide (TBX) agar with and without cefotaxime (6 mg L^−1^). All agars were incubated at 37±2 °C for 24 ± 2h after which distinct colonies were counted using image processing software (OpenCFU) (Geissmann, 2013).

**Treatment procedure:** resuspended *E. coli* and *E. faecium* pellets were spiked in 600 ml of urine and mixed for 30 s on a magnetic stirrer (Section 2.3). The urine was then poured down into the photoreactor. Once the photoreactor was placed in the cooling bath, the UV lamp was turned on to start the treatment. At start and end of each treatment period a 1 ml sample was taken for bacteria enumeration and 50 ml of urine was collected, mixed with 20% (v/v) of Tris-EDTA, and stored at −20 °C until use for ARG analysis. For the treatments involving hydrogen peroxide, 1.25 g H_2_O_2_ L^−1^ (36 mM) was added to the inoculated urine before pouring it down the photoreactor. Controls, were performed following the same procedure using the same photoreactor but without UV irradiation or hydrogen peroxide, to mimic the experimental condition, but studied for fewer time intervals (Table 1). Control samples were measured at selected intervals during the main experiment, based on the expectation of minimal variation under the tested conditions as indicated by pre-trial experiments. Additionally, post-trial measurements conducted at the final treatment time points (20 and 80 min) confirmed the stability of the control samples.

### 2.4 DNA extraction and qPCR

**DNA extraction and strain confirmation:** to quantify the abundance of *bla*_CTX − M_ and *van*-A gene, DNA was extracted from a 22 ml mixture of tris-EDTA and urine using DNeasy blood and tissue test kit (cat.no 69504, Qiagen, Germany) after equilibration of samples to room temperature. The DNA concentration was measured using Qubit^®^ 3.0 Fluorometer (life technologies, Malaysia). Confirmatory 16S-rRNA –based Sanger sequencing was run on the extracted DNA to check *E. coli* and *E. faecum* and rule out contamination (Supplementary Table 1).

**Resistant genes amplification, cloning and Plasmid DNA isolation**: The *bla*_CTX − M_ and *van*-A genes were PCR amplified, sequenced for confirmation and cloned in preparation for quantitative Real-Time PCR (qPCR)-based gene quantification. *bla*_CTX − M_ and *van*-A genes from the extracted DNA were amplified using the primers and reaction conditions in Table 2. Each PCR reaction (25 μl) contained 5 μl template DNA, 12.5 μl Taq polymerase, 1 μl of both forward and reverse primer and 5.5 μl water. Optimized PCR conditions listed in Table 2 were followed and the PCR product was quantified using Qubit^®^. The PCR product size was confirmed using gel electrophoresis (1% agarose in tris-EDTA).

**Table 2:** Primer, probe, size of the amplification product and optimized conditions of PCR and qPCR assays for antibiotic-resistant genes extracted from *E. coli* and *E. faecium*.

**Type of primer**		**Sequence (5^′^-3^′^)**	**Reference**
*bla*_CTX − M_	Forward	ATGTGCAGCACCAGTAAAGTGATGGC	Sittová et al., 2015
	Reverse	ATCACGCGGATCGCCCGGAAT	
	Probe	HEX-CAGCGGGTA/ZEN/CTCCTACCTGATT-3IABkFQ	
	Amplification product	336 bp	
	Optimized PCR conditions	95 °C for 10 min, 45 cycles of 15 sec at 95 °C, 40 sec at 64 °C, 40 sec at 72 °C, and 1 min at 72 °C	This study
	Optimized qPCR conditions	95 °C for 10 min, 45 cycles of 15 sec at 95 °C, 40 sec at 64 °C and 40 sec at 60 °C.	
*van*-A	Forward	GCCGGAAAAAGGCTCTGAA	He et al., 2020
	Reverse	TTTTTTGCCGTTTCCTGTATCC	
	Probe	FAM-CGCAGTTATAACCGTTCCCGCAGACC-BHQ1	
	Amplification product	90 bp	
	Optimized PCR conditions	95 °C for 10 min, 40 cycles of 15 sec at 95 °C, 40 sec at 57.5 °C, 40 sec at 72 °C, and 1 min at 72 °C	This study
	Optimized qPCR conditions	95 °C for 10 min, 45 cycles of 15 sec at 95 °C, 40 sec at 57.5 °C and 40 sec at 60 °C.	

Following the manufacturer's instructions, the PCR product was cloned to plasmid using pGEM^®^-T vectors (Promega GeneJET plasmid miniprep kit, Thermo Scientific, K0502) and transformed to JM 109 high efficiency competent *E. coli* cells overnight at 37 °C. Transformation was confirmed by plating the cells in 50 μg ml^−1^ ampicillin-containing agar plates. Plasmid DNA extraction was performed on transfromants grown overnight in LB broth as per the instructions in GeneJet purification kit (Thermo Scientific, K0702). Furthermore, the PCR product and plasmid DNA from transformed cells were sanger sequenced by Macrogen Europe (Netherlands) to confirm the amplification was from the intended gene of interest. The obtained sequence was nucleotide blasted on CARD database (Alcock et al., 2023) for confirmational purposes (Supplementary Table 2).

**Standard curve preparation and qPCR reaction:** The extracted Plasmid DNA were successively diluted to prepare a standard qPCR curve in the range of 10 to 10^8^. A qPCR reaction was performed using 96 well qPCR machine (QuantStudioTM 5 Real-Time PCR, applied biosystems, Thermo Fisher Scientific, USA). The built-in design and Analysis software 2.0 was used to design the plates, set the reaction condition, and collect the data. A triplicate of negative control was run in each qPCR as quality control. PCR conditions were optimized, following the protocols outlined in Table 2. The primers and probes used in this study were taken from previous publications (listed in Table 2), with modification. Each qPCR reaction (25 μl) contained 3 μl template DNA (1:100 diluted), 12.5 μl Maxima^TM^ probe qPCR master mix (Thermo scientific, K0261, USA), 1 μl of both forward and reverse primer, 0.6 μl probe and 6.9 μl nuclease free water. Quantitative PCR for *bla*_CTX − M_ and van-A genes were performed using the primers and probes listed in Table 2. The expected amplicon size was confirmed through agarose gel electrophoresis (Supplementary Figure 1).

### 2.5 Analysis of standard physico-chemical parameters

All the chemicals and reagents used in the study were of an analytical grade. For measurements of pH and EC, a pH electrode with an integrated Pt1000 temperature sensor (6.0258.010, Herisau, Switzerland) and an EC cell (6.0917.080, Metrohm, Herisau, Switzerland) connected to pH/EC meter (Metrohm, CH-9100 Herisau, Switzerland) were used. To adjust the pH of the urine, 5M KOH and 1 M H_2_SO_4_ were used. The UV absorbance of the urine was measured in the wavelength range of 190–400 nm using a Lambda 365 UV-vis spectrophotometer (Perkin-Elmer, United States) with 1 cm optical path length, prior to which urine samples were diluted 100-fold with Milli-Q water.

The concentration of total nitrogen (N_tot_), total ammonia nitrogen, and chemical oxygen demand (COD) was determined colorimetrically using Spectroquant^®^ test kits (Merck KGaA, Darmstadt, Germany) and a spectrophotometer (NOVA 60 A, Merck KgaA, Germany). COD measurements were adjusted following the method described by (Kang et al. 1999) to account for the potential interference of residual peroxide. The concentration of residual peroxide in urine was determined following the procedure described by (Arve and Popat 2021).

### 2.6 Data analysis

The bacteria inactivation, i.e., the reduction in bacteria concentrations, for each treatment period was given as -log_10_ cfu by normalizing end concentrations to start concentrations as log_10_ (C_t_/C_0_). Models for *E. coli* and *E. faecium* inactivation over time were fitted for each treatment by combining the normalized inactivation data for the different treatment times which were studied independently. Changes in log_10_ reduction over time was tested against two inactivation models, a log-linear model (Equation 1), and a model for shouldered inactivation curves (Equation 2) suggested for UV inactivation (Harm, 1980) and also used for chemical inactivation. Inactivation kinetics along with prediction interval (95% confidence limit), the latter when the number of data points allowed, (Minitab 15; Minitab Ltd., United Kingdom), was derived using Equations 1–3.


(1) $$\begin{array}{l} Log_{10}\left(C_{t}\right)=Log_{10}\left(C_{0}\right)+k^{*}t \end{array}$$

(2) $$\begin{array}{l} Log_{10}\left(C_{t}\right)=\;Log_{10}\left(C_{0}\right)-Log_{10}\left[1-{\left(1-10^{k^{*}t}\right)}^{10^{n}}\right] \end{array}$$

(3) $$\begin{array}{l} n=l^{*}|k| \end{array}$$

where *C*_0_, and *C*_*t*_ are concentrations of colony forming units at time zero and time t, *k* is first-order inactivation rate (min^−1^), *t* is time (min), and *n* is an empirical value which is used to calculate the lag period *l* (min). Both Equations 1, 2 were used to model reduction kinetics of ARGs.

Results from qPCR (cycles) were converted to gene copies using Equation 4.


(4) $$X_{o}={E_{amp}}^{\left(b-C_{q}\right)}=10^{\left(\frac{\left(C_{q}-b\right)}{m}\right)}$$

where *X*_*o*_ is gene copy number, E_amp_ is efficiency of amplification, C_q_ is the cycle number, m and b are the slope and the constant of the regression equation of the standard curve, respectively.

Treatments employing UV are also expressed in terms of incident fluence/UV dose and conversion of treatment time into UV dose is calculated according to Equation 5.


(5) $$\begin{array}{r} \text{UV dose }\left(\text{mJ c}{\text{m}}^{-2}\right)\;=\text{ Fluence rate }{\left(\text{mW c}{\text{m}}^{-2}\right)}^{*}\\ \text{treatment time }\left(\text{sec}\right) \end{array}$$

## 3 Results

### 3.1 Inactivation of antibiotic-resistant *E. coli* and *E. faecium* strains

**Figure 1:**
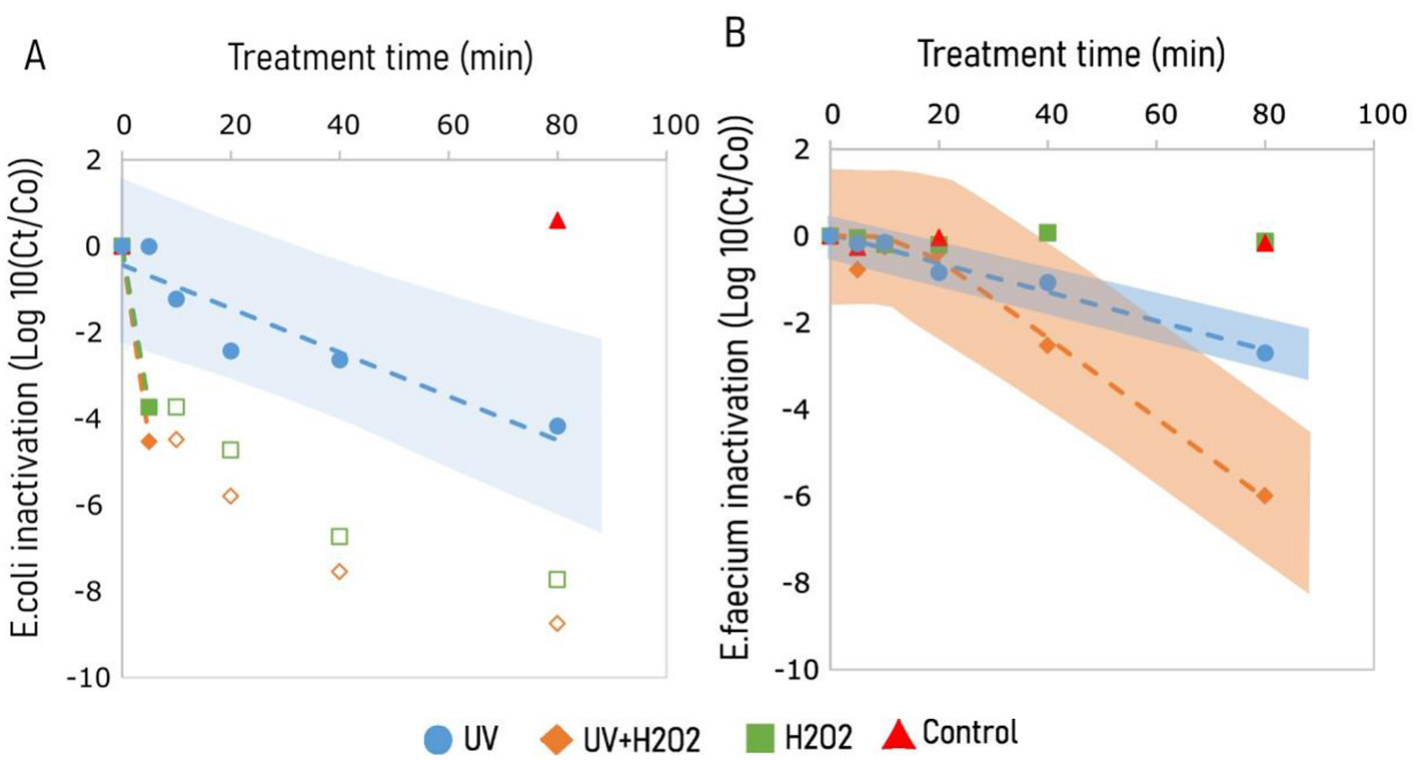
Inactivation of pathogens in KOH alkalized human urine (pH 10.8) exposed to treatments of UV, UV/H_2_O_2_, and H_2_O_2_, and control for β lactamase producing *E. coli* **(A)**, and Vancomycin resistant *E. faecium* **(B)**. UV irradiation was done using 65 W low pressure high output mercury lamps emitting light radiation at 185 and 254 nm. Experiments involving H_2_O_2_ treatment were dosed with 1.25 g H_2_O_2_ L^−1^. Hollow markers show plate count results that are below the detection limit. Inactivation kinetics were predicted using Equations 1, 2, represented by broken lines, *E. coli* (Blue) and *E. faecium* (orange). Shaded regions represent prediction interval for inactivation models for treatments of UV (blue) and UV/H_2_O_2_ (orange). The shaded area shows the 95% prediction interval derived from the fitted model and reflects uncertainty in parameter estimates, not experimental variation from replicated samples.

**Figure 2:**
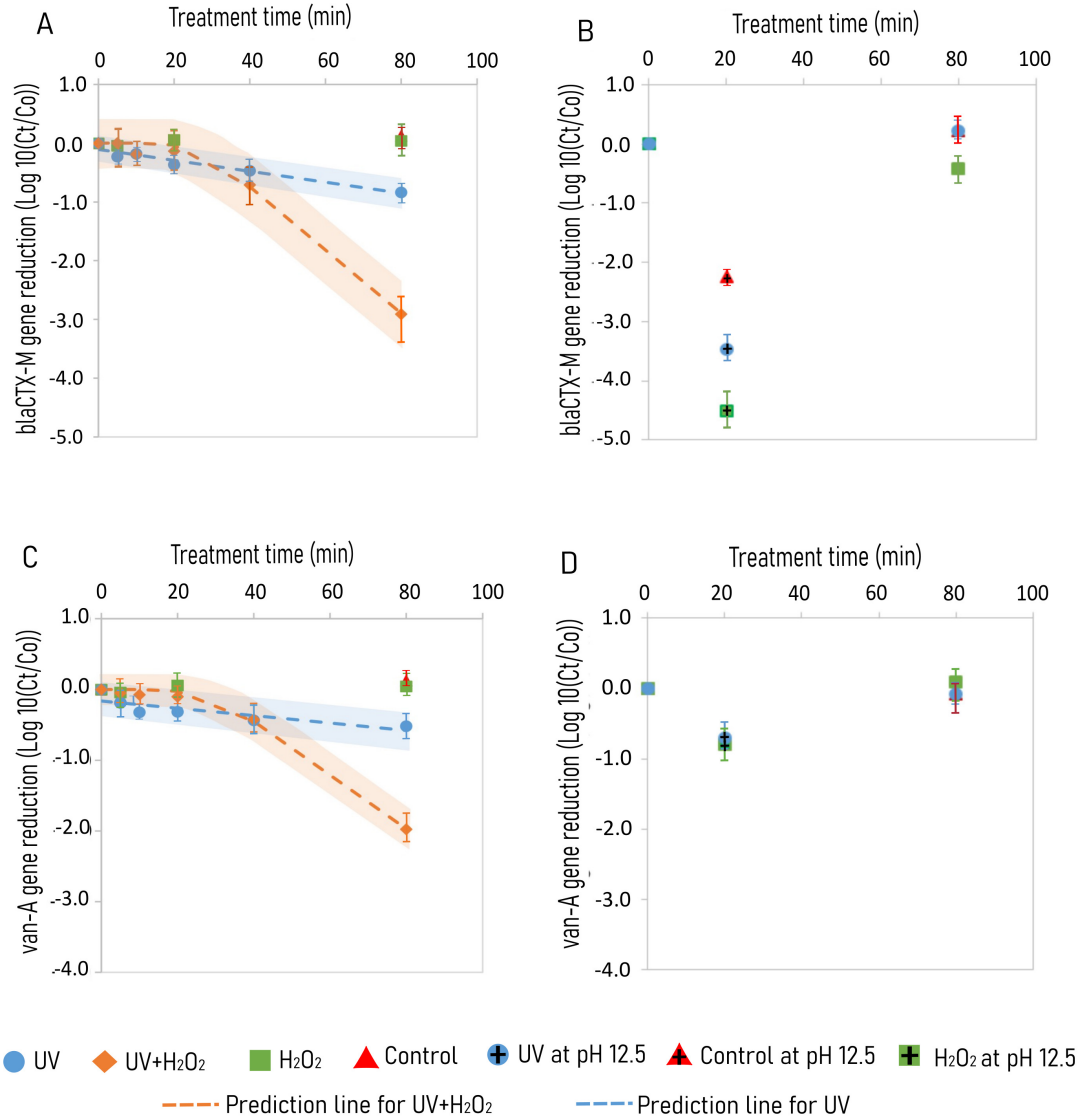
Degradation of ARGs in KOH alkalized urine subjected to treatments of UV, UV/H_2_O_2_, H_2_O_2_ and control for bla_CTX − M_ gene at pH 10.8 **(A)** and pH 7.0 and 12.5 **(B)**, and van-A gene at pH 10.8 **(C)** and at pH 7.0 and 12.5 **(D)**. Degradation kinetics are represented by broken lines with shaded regions showing prediction intervals for UV + H_2_O_2_ (orange) and UV (blue) treatment, respectively. UV irradiation is done using 65 W low pressure high output mercury lamps emitting photons at 185 and 254 nm. Samples involving H_2_O_2_ treatment were dosed with 1.25 g H_2_O_2_ L^−1^. Standard deviations are color coded inaccordance with the treatment type.

Plating *E. coli* on a cefotaxime containing plate after 80 min treatment revealed the inactivation of *E. coli* with 4 log_10_ for UV, and more than 6.5 and 7.5 log_10_ for H_2_O_2_ and UV/H_2_O_2_ treatment, respectively (Figure 1A). All three treatments resulted in a higher inactivation of *E. coli* compared to the control (without UV and H_2_O_2_), which exhibited only a 0.5 log_10_ inactivation over 80 min at the initial pH 10.8 (Figure 2A). *E. coli* was inactivated with treatment by UV following a log-linear inactivation with an inactivation rate constant (*k)* of −0.06 log_10_ cfu min^−1^ (Table 3). At 5^th^ min, treatments of H_2_O_2_ and UV/H_2_O_2_ had *E. coli* concentrations below the detection limit, indicating an inactivation of more than 1 log_10_ cfu min^−1^ (Figures 1A,B).

**Table 3:** Inactivation kinetics for *E. coli* and *E. faecium* and antibiotic resistant genes, bla_*CTX*−*M*_ and van-A gene under treatments of UV, H_2_O_2_ and UV/H_2_O_2_ in KOH alkalized urine at pH 10.8.

	**Test organism**	**Treatment type**	***k*- Value ±Std.err (log_10_ cfu or GC min^−1^)**	**Type of model fit**	**Lag time (min)**	**Model fit (*R*^2^)**
ARB	*E. coli*	UV	−0.058 ± 0.0075	Log-Linear	–	0.82
		UV/H_2_O_2_	< –1^*^	Log-Linear	–	–
		H_2_O_2_	< –1^*^	Log-Linear	–	–
	*E. faecium*	UV	−0.033 ± 0.0017	Log-Linear	-	0.97
		UV/H_2_O_2_	−0.093 ± 0.0097	Lag+Log linear	15	0.98
		H_2_O_2_		No inactivation		
ARG	*bla*_CTX − M_	UV	−0.0093 ± 0.0013	Log-Linear	–	0.94
		UV/H_2_O_2_	−0.055 ± 0.0032	Lag+Log linear	27	0.99
		H_2_O_2_		No reduction		
	*van*-A	UV	−0.0052 ± 0.0011	Log-Linear	–	0.72
		UV/H_2_O_2_	−0.044 ± 0.0019	Lag+Log linear	31	0.99
		H_2_O_2_		No reduction		

(*) Refers to the *k* values calculated based on detection limits being met in 5 min. ARB and ARG refers to the antibiotic-resistant bacteria and the antibiotic-resistant gene, respectively. GC and cfu refers to gene copies and colony forming units respectively.

Inactivation of *E. faecium* was observed for treatments of UV and UV+H_2_O_2_ with a 2.7 log_10_ and a 6 log_10_ inactivation in 80 minutes, respectively (Figure 1B). However, treatment of H_2_O_2_ alone had no effect on the inactivation of *E. faecium*. Unlike UV treatment, inactivation of *E. faecium* under treatments of UV/H_2_O_2_ had a lag phase of 15 min (Table 3). For *E. faecium*, inactivation rate constants of −0.033 and, −0.093 log_10_ cfu min^−1^ were observed for treatments of UV and UV/H_2_O_2_, respectively (Table 3). Inactivation for *E. faecium* was faster when UV treatment was combined with H_2_O_2_ (Figures 1A,B).

For treatment at pH 7.0, both UV and H_2_O_2_ achieved 3 log_10_ inactivation of *E. coli* within the 80 min treatment, while no inactivation was observed for the control measurements (without UV and H_2_O_2_). However, for *E. faecium*, inactivation at pH 7.0 was observed only with UV treatment, with a 1.5 log_10_ inactivation in 80 min. For UV and H_2_O_2_ treatments at pH 12.5, inactivation beyond the detection limit was observed within 20 min of treatment; >7 log_10_ and >3 log_10_ inactivation for *E. coli* and *E. faecium*, respectively. The same inactivation was observed after 20 min in control (without UV and H_2_O_2_) at pH 12.5. A post-trial experiment conducted using the same KOH alkalized urine showed more than 7 log_10_ inactivation for *E. coli* within 1 min of exposure to a pH of 12.5 (data not shown). Bacterial inactivation results indicate a clear trend over time, it is important to note that the data were generated using a single-replicate design without repeated measurements at each time point. Consequently, the precision of individual data points is limited, hence observed trends require careful interpretation.

### 3.2 Degradation of antibiotic-resistant genes

Recombinant plasmid DNA carrying either *bla*_CTX − M_ or *van*-A gene was amplified and visualized by gel-electrophoresis and the results showed DNA fragments in accordance with the expected PCR product length (Supplementary Figure 1). Confirmatory sequencing of the plasmid insertions revealed a 100% identity match for *van*-A gene in *E. faecium* (ARO:3000010) and 100% identity match for *bla*_CTX − M_ gene in *E. coli* (ARO:3005661) when blasted against reference sequences on CARD database (Alcock et al., 2023). The efficiency of the qPCR reaction for plasmid standard curves of each respective gene were in the range of 95 and 105%, and *R*^2^ of 0.99.

With the 80 min treatment of urine at an initial pH of 10.8, a 1 and 3 log_10_ reduction of the *bla*_CTX − M_ gene copy was observed for UV and UV/H_2_O_2_ treatment, respectively (Figure 2A). Treatment with H_2_O_2_ alone and control without UV and H_2_O_2_, revealed no reduction of both *bla*_CTX − M_ and *van*-A gene over 80 min treatment (Figure 2). The *bla*_CTX − M_ gene showed a log-linear reduction with a *k* value of −0.009 ± 0.001 and −0.055 ± 0.003 log_10_ gene copies min^−1^ for treatments of UV and UV/H_2_O_2_, respectively (Table 3). Degradation of *van*-A gene was also observed for treatments of UV and UV/H_2_O_2_ (Figure 2C). Treatment of UV/H_2_O_2_ for 80 min resulted in 2 log_10_ gene copy reduction with the *k* value of −0.04 log_10_ gene copies min^−1^ with a lag time of 31 and 80 min treatment with UV resulted in a 0.5 log_10_ gene copy reduction with a *k* value of −0.0052 log_10_ gene copies min^−1^ (Figure 2C, Table 3).

For 20 min treatment at pH 12.5, the *bla*_CTX − M_ gene was reduced with 2.2, 3.5, and 4.5 log_10_ gene copies for control, and treatments of UV and H_2_O_2_, respectively (Figure 2B). However, among the 80 min treatments at pH 7.0, gene copy reduction was observed only for H_2_O_2_ with 0.5 log_10_ gene copies (Figure 2B). Reduction of *van*-A gene occurred only at pH 12.5 with a comparable gene reduction of 1 log_10_ for control and treatments of UV and H_2_O_2_ (Figure 2D).

## 4 Discussion

### 4.1 Inactivation of antibiotic-resistant bacteria

Treatment of alkalized urine (pH 10.8) for 80 min with H_2_O_2_ and UV/H_2_O_2_ resulted in more than 6 log_10_ inactivation for both *E. coli* and *E. faecium*. However, inactivation of the two organisms differed for the H_2_O_2_ treatment, i.e., *E. coli* was inactivated with more than 6 log_10_ while *E. faecium* showed persistence toward treatments of H_2_O_2_ (1.25 g L^−1^) with only 0.01 ± 0.08 log_10_ inactivation. The reduction of *E. coli* is in line with earlier studies, e.g., 30 mM H_2_O_2_ gave >6 log_10_ *E. coli* reduction in citric acid-Na_2_HPO_4_ buffer solution under 5 min (Raffellini et al., 2011) while 0.3 mM H_2_O_2_ resulted in no reduction of *E. coli* in phosphate buffer solution within 30 min (Sun et al., 2016). The high dose of H_2_O_2_ (37 mM) in this study could be one of the reasons for the higher inactivation of *E. coli* as compared to previous studies.

For treatments involving reactive oxygen species (H_2_O_2_, OH^*^) to inactivate microorganisms, the first step in the process is the damage of cell walls. *E. faecium*, a gram positive bacterium, has a thicker cell wall (25 nm; Mishra et al., 2012) compared to *E. coli* (4 nm; Gan et al., 2008). Therefore, the underlying difference in cell structure could explain the fast inactivation of *E. coli* while *E. faecium* concentrations were not decreased by H_2_O_2_ treatment alone (Figure 1) (Zhang et al., 2023; Rodríguez-Chueca et al., 2015). Moreover, exposure to H_2_O_2_ has the ability to modify cell surface charge of Gram-positive bacteria which leads to an aggregation of bacteria, thus retarding treatment efficacy of microbial inactivation (Zhang et al., 2023). In contrast to Gram-positive bacteria, Gram-negative bacteria, such as *E. coli*, does not undergo such a change in surface charge upon exposure to oxidants, such as H_2_O_2_, thus restricting cell aggregation. This allows a free interaction of the oxidant with the cell (Zhang et al., 2023). Such a phenomenon could explain the observed lag phase for *E. faecium* when exposed to the combination of UV + H_2_O_2_ (Figure 1B), which resulted in an immediate inactivation of *E. coli* (Figure 1A).

Inactivation studies employing H_2_O_2_ treatment coupled with pH have demonstrated that both high pH (pH 9; Batterman et al., 2001) and low pH (pH 3; Raffellini et al., 2011) results in enhanced inactivation (>2 log_10_ higher inactivation) compared to the neutral pH. This may explain the observed difference in inactivation of both bacteria to pH controls between pH 10.8 and 12.5 (Figure 1). However, the inactivation of both bacteria in urine alkalized to a pH 12.5 was so rapid that any added effect of H_2_O_2_ or UV could not be observed (Figure 1).

In this study an inactivation of 2.7 and 1.5 log_10_ was observed for UV treatment at pH 7.0 for *E. coli* and *E. faecium*, respectively. (Hokanson et al. 2016) showed *E. coli* to have a higher susceptibility to photolysis, with a photolysis coefficient of 238,593 L Einstein^−1^ cm^−1^, compared to *E. faecalis*, with a coefficient of 147,116 L Einstein^−1^ ${\text{cm}}_{,}^{-1}$ which may explain the higher inactivation rate of *E. coli* compared to *E. faecium* in this study. (Mckinney and Pruden 2012) studied the inactivation of antibiotic resistant *E. faecium* (*van*-A) and *E. coli* (*tet*(A)) using UV in filtered wastewater. The authors reported that for a 3 log_10_ inactivation *E. faecium* required an at least 2-fold higher UV dose compared to *E. coli*, similar to what was observed in this study. Conversely, a study by (He et al. 2021) claimed that G+ bacteria, such as *E. faecalis*, are more susceptible for photocatalysis than G- bacteria, such as *E. coli*, indicating that susceptibility toward UV treatment cannot be generalized by the gram features of the cell. A review by (Hijnen et al. 2006) also stated that the sensitivity toward UV treatment differs between different strains of the same species. Additionally, *E. faecium* occurs in pairs or chains and enterococci in general are prone to clustering leading to less exposure to UV, which can explain the demoted inactivation compared to *E. coli* due to UV and H_2_O_2_ treatment in the present study.

Studies conducted using monochromatic low pressure mercury lamp (254 nm) reported that up to 15 mJ cm^−2^ UV dose and 20 mJ cm^−2^ is required for >5 log_10_ inactivation of *E. coli* in deionized water (Harris et al., 1987) and secondary treatment effluent, respectively (Nasser et al., 2006). However, in this study UV doses of 880 mJ cm^−2^ and < 55 mJ cm^−2^ were required for 4 log_10_ inactivation of *E. coli* with UV alone and UV/H_2_O_2_ treatment at pH 10.8, respectively (Figure 1). Additionally, (Hokanson et al. 2016) reported an inactivation *k* value of −0.506 cm^2^ mJ^−1^ for *E. coli* under UV treatment (254 nm) in water. In this study *E. coli* was inactivated in KOH alkalized urine at pH 10.8 with a *k* value of −0.0053 cm^2^ mJ^−1^ and −0.0824 cm^2^ mJ^−1^ for treatments with UV and UV/H_2_O_2_, respectively (Supplementary Table 3). However, the exposure to a lower wavelength UV light has an increased effectiveness toward microbial inactivation as it can cause comparatively high damage compared to UV 254 nm, which can explain the observed difference (Clauß, 2006). Indeed, (Clauß 2006) studied microbial inactivation using a krypton-chloride excimer lamp emitting photon at 222 nm and a LP mercury lamp at 254 nm and reported UV treatment at 222 nm resulting in comparatively higher inactivation compared to treatment at 254 nm. Additionally, (Moussavi et al. 2019) reported that vacuum UV (185 + 254 nm) treatment resulted in 2-fold inactivation of *E. coli* compared to UV 254 nm when given the same treatment conditions. Furthermore, (Giannakis et al. 2018) reported a >4 log_10_ *E. coli* inactivation in real urine for a treatment time of 45 min by UV (254 nm) at pH 7.0, contrasting to the present study in which only 3 log_10_ *E. co*li inactivation was achieved with 80 min of UV treatment at pH 7.0, even when the lamp we used emitted light at both 185 and 254 nm. A possible explanation for these results is that the effect of a lower wavelength (185 nm) is overshadowed by the presence of organic matters in urine that has high UV absorbance at this wavelength (Demissie et al., 2024).

Inactivation studies conducted in wastewater effluent requires a higher UV dose when compared with pure water or phosphate solution (Hijnen et al., 2006). The study by (Giannakis et al. 2018) revealed that >4-fold higher treatment time was required for comparable *E. coli* inactivation in real urine (>45 min) compared to activated sludge effluent water ( ≤ 10 min). Depending on the organisms intended to be removed, it may require even higher doses. For example, a UV dose of 400 mJ cm^−2^ was required for 2.5 log_10_ inactivation of *Ascaris suum* eggs in phosphate buffer saline solution and 560 mJ cm^−2^ for >4 log_10_ inactivation of *Aspergillus niger* spores in demineralized water (Brownell and Nelson, 2006; Clauß, 2006; Masjoudi et al., 2021). Microbial particle association, light interference, and scavenging properties of the matrix are possible reasons for the requirement of high treatment time/UV dose for inactivation in wastewater and urine solutions (Giannakis et al., 2018; Örmeci and Linden, 2002). On the contrary, inactivation studies employing a combination of UV/H_2_O_2_ show that treatment time significantly decreases with an increase in H_2_O_2_ dose. UV activates H_2_O_2_ by resulting in two OH^*^ which are non-selective oxidants that enhance microbial inactivation (Vilhunen et al., 2011; Giannakis et al., 2018), which likely explains the higher observed inactivation of *E. faecium* during treatment UV/H_2_O_2_ compared to treatments of UV and H_2_O_2_ alone (Figure 1B).

Aside from pH 12.5, controls without UV and H_2_O_2_ at pH 7.0 as well as 10.8 did not result in any inactivation of both test organisms (Figure 2). *E. faecalis*, a close relative of *E. faecium*, is reported to survive high pH (pH 11), however, microbial growth was highly affected for pH >11.5 (Mchugh et al., 2004; Starliper and Watten, 2013). High alkaline pH inactivated bacteria through the action of hydroxyl anions. Hydroxyl anions have the ability to (i) damaging cytoplasmic membrane, (ii) denature enzymes and (iii) damage DNA (Siqueira Jr and Lopes, 1999). This therefore explains the fast inactivation of both test organisms at pH 12.5.

### 4.2 Reduction of antibiotic-resistant genes

In this study, up to 3 log_10_ gene reduction was observed for *bla*_CTX − M_ with treatments of UV/H_2_O_2_ whereas only 2 log_10_ was noted for *van-A* gene with the same treatment and time (80 min ≈ 880 mJ cm^−2^; Figure 2). Amplicon size is reported as one of the factors for the indifference in degradation rate of genes as there are fewer pyrimidine dimer (TT,CT,TC or CC) targets as the gene size gets shorter (He et al., 2022; Mckinney and Pruden, 2012). (He et al. 2019) studied degradation of extracellular blt gene with amplicon sizes ranging from 266 bp to 1017 bp and reported that there is a 4-fold increase in gene degradation during UV treatment at pH 7.0 for the large amplicon size (−0.12 cm^2^ mJ^−1^, 1017 bp) compared to the smaller amplicon size (−0.025 cm^2^ mJ^−1^, 266 bp). The relative difference in amplicon size could explain the relatively higher degradation of *bla*_*CTX*−*M*_ gene (336 bp) and *van-A* gene (96 bp) in the present study (Table 2).

According to a review by (Han et al. 2023), a comparatively higher UV dose is required to degrade ARGs rather than inactivate ARBs. This is in line with the results in the present study which shows a difference in inactivation of ARBs (>6 log_10_) with degradation of ARGs ( ≤ 4 log_10_) (Figures 1, 2). Interferences of matrix, formation of cell clusters, and scavenging of both photons and oxidants formed during UV irradiation by lysed cell matters may contribute to the demoted degradation of ARGs compared to the inactivation of ARBs. In this study, a UV treatment time of 80 min (which is equivalent to 880 mJ cm^−2^) was required to degrade a 96 bp *van*-A gene by 0.5 log_10_ gene copies min^−1^. Notably, (Mckinney and Pruden 2012) reported a UV dose (254 nm) of 200 mJ cm^−2^ to be required for 4 log_10_ reduction of both intracellular and extracellular *van*-A (1,030 bp) gene in phosphate buffer solution.

During treatments of urine with UV, sulfate, phosphate, and carbonate-radicals are formed in addition to the hydroxyl radicals (Zhang et al., 2016, 2015). These radicals react with DNA bases with a rate constant of 5 × 10^7^ −9 × 10^9^ L mol^−1^ s^−1^. For example, thiamine reacts with 2.1 and 1.1 × 10^9^ L mol^−1^ s^−1^ with ${\text{SO}}_{4}^{-*}$ and ${\text{PO}}_{4}^{2-*}$, respectively (Ma et al., 2018). Therefore, such radicals might also be involved in the reduction of *bla*_CTX − M_ and *van–A* gene during UV irradiation of urine.

Hydrogen peroxide, however, does not damage DNA directly but rather through the production of hydroxyl radicals reacting with iron containing molecules (Mendoza-Chamizo et al., 2018). Hydroxyl radicals react with nucleotide bases to a create single lesion on DNA (Cadet and Wagner, 2013). For instance, thiamine reacts with OH^*^ with a rate 7.4 × 10^9^ L mol^−1^ s^−1^ (Ma et al., 2018). This explains the higher reduction of ARGs by UV/H_2_O_2_ treatment compared to UV alone (Figures 2A,C). Further, with a UV dose of 600 mJ cm^−2^ by the low pressure UV lamp (254 nm) around 10^14^ M OH^*^ are formed in a solution containing 0.3 mM H_2_O_2_ (Rosenfeldt et al., 2006). Thus, the increased formation of OH^*^ for experiments involving a combination of H_2_O_2_ and UV explains the higher reduction of ARG compared to UV alone (Figures 2A,C). However, treatment of H_2_O_2_ resulted in higher reduction of *bla*_CTX − M_ gene compared to UV treatment at pH 12.5 (Figure 2D). As explained in section 4.1, gram-negative bacteria is more susceptible to H_2_O_2_ treatment compared to gram-positive ones (Zhang et al., 2023). Consequently, DNA of *E. coli* will be more exposed to H_2_O_2_ action when compared to DNA of *E. faecium*, which is protected by cell aggregates. This explains the higher reduction of *bla*_CTX − M_ gene compared to *van*-A gene in treatments at pH 12.5 (Figures 2B,D).

High pH (pH > 11) inactivates pathogens through disruption of cytoplasmic membrane which leads to cell lysis and the release of DNA to the solution (Mendonca et al., 1994). At pH > 11, DNA is denatured and becomes single stranded by abstraction of hydrogen by OH ion (England et al., 2021; Bivehed et al., 2023). Gram-negative microorganisms are more prone to cell lysis at pH > 11 compared to gram-positive microorganisms. This also explains the comparative ARG reduction difference between *bla*_CTX − M_ and *van*-A gene with control treatment at pH 12.5. (Goetsch et al. 2020) reported 2 log_10_ reduction in the transformation efficiency of extracellular plasmid DNA harboring ampicillin and tetracycline resistant gene incubated in hydrolyzed urine for 24 h. Therefore, DNA damage for treatments at pH 12.5 could be greater when considering transformation efficiency of ARGs downstream urine processing steps. For instance, urine dehydration, one of the nutrient concentration process steps following urine stabilization, could be done with a temperature reaching 60 °C (Simha et al., 2020) and enzymes or proteins responsible for DNA repair could be denatured at this temperature which could further decrease the chance of ARG transfer (Boulon et al., 2010).

The results of inactivation of ARBs and degradation of ARGs indicate that it takes a comparably longer treatment time, or UV dose, for degradation of ARGs compared to ARBs, which is in line with previous studies conducted in water and wastewater matrices (Hokanson et al., 2016; He et al., 2022). However, comparisons between bacterial inactivation and ARG degradation results should be interpreted with caution, as the inactivation data were obtained from a single-replicate design without repeated measurements at each time point. Increasing the pH to 12.5 resulted in increased inactivation and degradation of ARGs to a level in which the use of UV or H_2_O_2_ treatment was not necessary. Thus, nutrient recovery technologies aimed at recovering N in the form urea-N could employ either a combination of UV/H_2_O_2_ treatment at pH 10.8 or prolonged storage time (>3 h) at pH 12.5 for enhanced 6 log_10_ inactivation of pathogenic bacteria and degradation of ARGs (>4 log_10_ gene copy), thereby keeping the nutrient potential intact (Supplementary Figure 2). Furthermore, considering UV/H_2_O_2_ treatment as pre-treatment for the recovery and use of nutrients from source separated urine, an 80 min treatment at pH 10.8 was sufficient to meet the performance target of 6 log_10_ microbial reduction set for unrestricted use of excreta for agricultural purposes (WHO, 2006). However, since the results presented here are based on laboratory conditions, further investigation is required for its applicability in decentralized source separation systems. (Demissie 2023) discussed the limitations of such technology for source separated urine and it's recommended for future studies to address such limitations.

## 5 Conclusion

This study investigated the inactivation of ARBs and reduction of ARGs in KOH alkalized urine (pH 10.8) subjected to treatments of UV, H_2_O_2_, and UV/H_2_O_2_. Compared to treatments of separate UV or H_2_O_2_, treatment with UV/H_2_O_2_ combined showed higher efficiency by inactivating ARBs and degradation of ARGs. UV/H_2_O_2_ treatment resulted in 3 log_10_ and 2 log_10_ reduction for *bla*_CTX − M_ gene and *van-A* gene, respectively. However, H_2_O_2_ alone did not have any effect on gene degradation but contributed when combined with UV, compared to UV alone. A 10-fold treatment time/UV dose was needed to achieve the same reduction in ARGs as in ARBs. A reduction rate constant of −0.055 and −0.04 log_10_ gene copies min^−1^ was observed for *bla*_CTX − M_ and *van-A* gene, respectively, under treatment of UV/H_2_O_2_ at pH 10.8, and reduction rates were a magnitude slower for UV alone. Treatment at pH 7.0 gave no reduction of ARGs and inactivation of ARBs were very low. Treatment of KOH alkalized urine at pH 12.5 resulted in faster inactivation of both ARBs and higher degradation of *bla*_CTX − M_ gene than treatments of urine at pH 10.8. Treatment of source separated urine with UV/H_2_O_2_ at pH 10.8 or storage (>3 h) at high pH (12.5) will reduce the potential risk of ARB and ARG dissemination during use of urine or urine derived fertilizer.

## Data Availability

The original contributions presented in the study are included in the article/Supplementary material, further inquiries can be directed to the corresponding authors.
