# Supplementary material for: Simultaneous inactivation of antibiotic-resistant bacteria and degradation of antibiotic-resistant genes in alkalised human urine
Source: Front Microbiol. 2025 Aug 22;16:1605625. doi: 10.3389/fmicb.2025.1605625 (PMC12411533; doi:10.3389/fmicb.2025.1605625)
Supplement: Supplementary file 1 [file Table_1.docx]

# Supplementary information

Simultaneous inactivation of antibiotic-resistant bacteria and degradation of antibiotic-resistant genes in alkalised human urine

**Natnael Demissie^a,b^, , Annika Nordin^a^, Prithvi Simha^a^, Isis Conroy^c^, He Sun^d^**, **Anna Schnürer^d^, Björn Vinnerås^a^, Adey Desta^b,e^**

***^a^****Department of Energy and Technology, Swedish University of Agricultural Sciences, Box 7032, SE–750 07 Uppsala, Sweden.*

*^b^Institute of Biotechnology, College of Natural and Computational Sciences, Addis Ababa University, Box 1176 Addis Ababa, Ethiopia.*

*^c^Department of Environmental Engineering, Tampere University of Applied Sciences, Kuntokatu 3, FI-33520 Tampere, Finland*

*^d^Department of Molecular Sciences, Swedish University of Agricultural Sciences, Uppsala, Box 7015, SE-750 07 Uppsala, Sweden.*

*^e^Department of Microbial, Cellular and Molecular Biology, College of Natural and Computational Sciences, Addis Ababa University, Box 1176 Addis Ababa, Ethiopia.*

^a,b^ Corresponding Author. Phone: +251913266138

e.mail: [natnael.demissie@slu.se](mailto:natnael.demissie@slu.se); adey.feleke@aau.edu.et

The primers used were specially targeting bacterial DNA as described by Edwards et al. (1989) (Table S1). DNA from other origin might have been present but were not targeted by the used method.

Table S1, type of primers used for detection of bacterial DNA

| Primer type | **Sequence** |
| --- | --- |
| Forward primer | 5'-AGAGTTTGACCTGGCTCAG-3' |
| Reverse primer 1522R | 5'-AAGGAGGTGATCCAGCCGCA-3' |


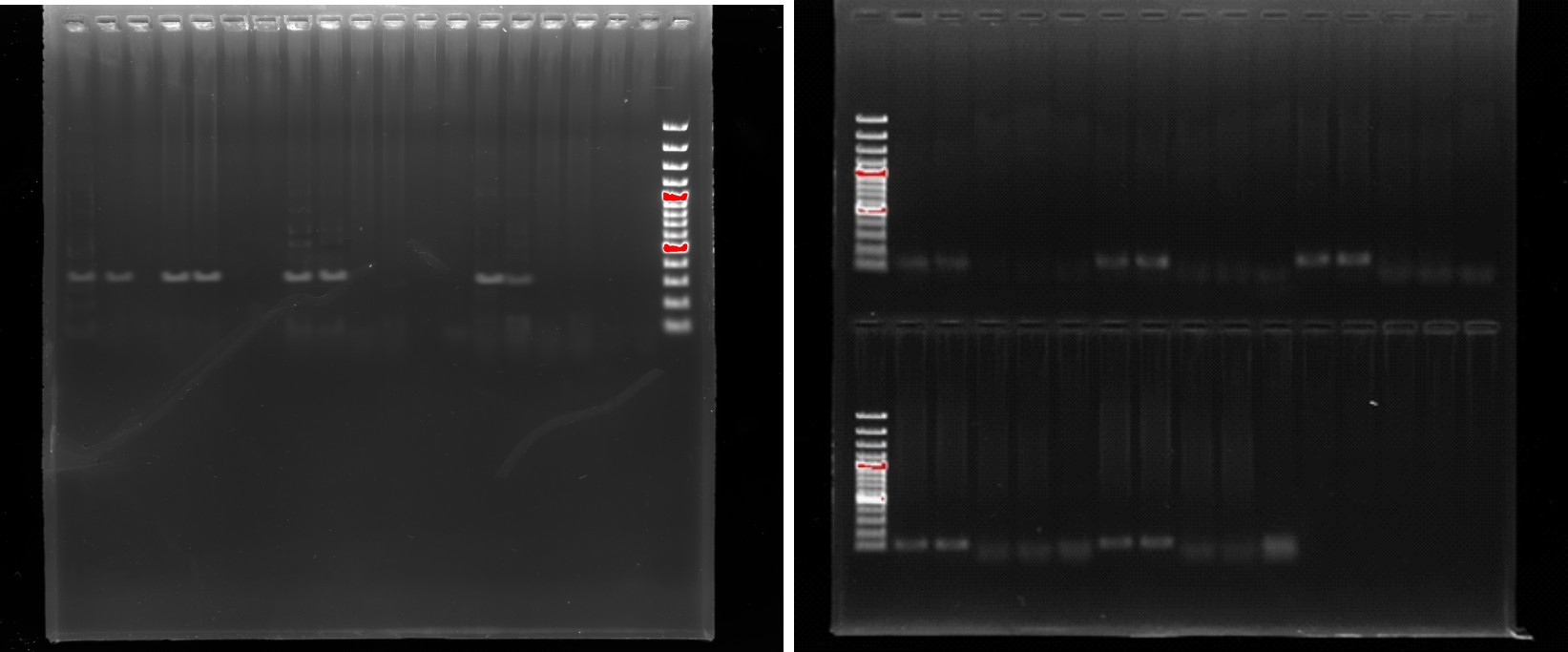


(A) (B)

Figure S1. Gel doc image for PCR amplification product for *bla*_CTX-M_ gene (A) and Van-A (B) gene with a product length of 96 bp and 336 bp respectively. Amplification was carried out by using the strains independently, meaning that the absence of amplification from a non-target organism was confirmed.

Table S2. Identity match for nucleotide blasted plasmid DNA carrying either of *bla*_CTX-M_ or Van-A gene in CARD data base

| **Gene type** | **Identity** | **Species** |
| --- | --- | --- |
| ***bla*_CTX-M_** | 100% | *Escherichia coli* |
|  | 100% | *Proteus mirabilis* |
|  | 100% | *Klebsiella pneumoniae* |
|  | 100% | *Shigella sp. SH219* |
|  | 100% | *Citrobacter freundii* |
|  | 100% | *Enterobacter cloacae* |
|  | 100% | *Enterobacter kobei* |
| ***van*-A** | 100% | *Enterococcus faecium* |

Figure S2. Concentration of Total-Nitrogen and Urea-N subjected to 80 minutes of control, UV, H_2_O_2_ and UV+H_2_O_2_ treatments at different pH. UV irradiation was conducted using 65 W high pressure dichromatic mercury lamp. Samples involving H_2_O_2_ treatment were dosed with 1.25 g H_2_O_2_ L^-1^.

Table S3. Inactivation kinteics of CTX-M1 resistant E.coli and Van-A resistant E.faecium under different treatment in KOH alkalised urine at pH 10.8 fitted against inactivation models (Eq 1 and 2)

|  | **Test organism** | **Treatment type** | **K- Value (cm^2^ mJ^-1^)** | **Type of model fit** | **Model fit (R^2^)** |
| --- | --- | --- | --- | --- | --- |
| **ARB** | E.coli | UV | -0.0053 | Log-Linear | 0.956 |
|  |  | UV+H_2_O_2_ | <-0.0824* | Log-Linear | - |
|  |  | H_2_O_2_ | <0.0679* | Log-linear | - |
|  | E.feacium | UV | -0.003 | Log-Linear | 0.975 |
|  |  | UV+H_2_O_2_ | -0.0063 | Log-Linear | 0.937 |
| **ARG** | CTX-M1 | UV | -0.001 | Log-Linear | 0.859 |
|  |  | UV+H_2_O_2_ | -0.0028 | Log-Linear | 0.884 |
|  | Van-A | UV | -0.0007 | Log-Linear | 0.72 |
|  |  | UV+H_2_O_2_ | -0.0019 | Log-Linear | 0.871 |

(*) Refers to the *k* values calculated based on inactivation for the first 5 minutes. ARB and ARG refers to the antibiotic-resistant bacteria and the antibiotic-resistant gene, respectively.
